# Health Tract, No. 251

**Published:** 1866-06

**Authors:** 


HEALTH TRACT, NO. 251.

### TRICHINIASIS.

All hatless and shoeless, with foxy hair and shirt sleeves shivering in the wind, a countryman gallopped into New Haven, exclaiming at the top of his voice "The Oliver Ellsworth has boiled his buster! The Oliver Ellsworth has boiled his buster"!! the steamboat of that name having exploded a few miles from town causing great havoc of life and limb, and this messenger was sent for aid for the wounded and dying! So with newspaper writers about the "pork disease," in the tumult of their minds they have run away, and not knowing what they say, have wrought consternations dire in nervous wives in the city; while thrifty dames in the country have emptied their snow-white lard pots into the river, and thrown their delightful smoked hams to the dogs. It always breeds mischief to run away with half a fact, especially if it is a practical one. A parcel of thick headed Dutchmen in the father-land, too lazy to cook their sausages, have impregnated their blood with myriads of animalcules, which imbedding themselves in the flesh, propagate with amazing fecundity and the body is eaten up piecemeal and alive, by worms, scarcely larger than a human hair, the person dying in excruciating torture; whereupon these hair-brained youngsters of the press under the pressure of lager and gin slings would persuade the people that "Death is in the pot" of pork, inevitably and under all circumstances; completely ignoring two important facts, that

1st. Not a dozen authenticated cases have ever occured in the U. States.

2d. Only those who eat *raw* pork suffer from the disease.

Any one who is too lazy to cook his pork sausages ought to be wormy; he ought to be imbedded in fleas for the compulsive exercise of vigorous scratching.

It argues a brain all void of thought,to suppose that a microscopic insect could survive a two or three hours boiling, or exist in a frying pan, hot enough to blister an elephant.

No doubt the water cure people are in rhapsodies with a "Told you so," as they have been insisting with all the power of demonstrative, bare assertion, reiterated the millionth time, that pork was poison; that it bred all the scrofula in the world, and that if its consumption as food were persisted in, the race would, at no distant day become extinct, all but themselves. As pork has been the main stay of the nation for hundreds of years, and statistics tell us, the average duration of human is life steadily increasing, we would advise the people to eat as much "ham and eggs" as heretofore, not to discard "Pork and Beans," to revel in sausages in their proper season, to supply themselves with a good store of hogs lard, every autumn for the years' use, and dismiss all apprehension of being eaten up alive by pig-worms; but always cook these articles most thoroughly.

